# The relationship between physical activity and subjective well-being in Chinese university students: the mediating roles of perceived health, social support and self-esteem

**DOI:** 10.3389/fspor.2023.1280404

**Published:** 2023-10-25

**Authors:** Tianzhi Liao, Yujia Yin, Xiaoyong Hu, Saizhao Tang, Yunsik Shim

**Affiliations:** ^1^ Department of Sports Science, Guiyang University, Guiyang, China; ^2^ Department of Sports Sociology, Guiyang University, Guiyang, China; ^3^ Department of Physical Education, Guiyang University, Guiyang, China; ^4^ Department of Sports Medicine, Guiyang University, Guiyang, China; ^5^ Department of Sports Science, Soonchunhyang University, Asan, Republic of Korea

**Keywords:** physical activity, subjective well-being, social support, perceived health, self-esteem

## Abstract

**Purpose:**

The intent of this paper is to understand the effect of Physical Activity on university students' Subjective Well-being and to explore whether Perceived Health, Social Support, and Self-esteem play roles as mediating variables.

**Methods:**

Self-reported data from 404 college students (147 males and 257 females) were analyzed using structural equation modeling (SEM). The relationships between the study variables were tested by mediation models and 5,000 bootstrap samples using AMOS version 24.

**Results:**

(1) The six hypotheses were supported in the measurement model in the results (*P* < 0.05). Physical Activity was related to Social Support, Perceived Health, and to Self-esteem; Social Support, Perceived Health, and Self-esteem were all related to Subjective Well-being. However, the direct positive effect of Physical Activity gradually decreased in the order of Self-esteem, Social Support, and Perceived Health. The direct effect of Perceived Health, Social Support, and Self-esteem on Subjective Well-being also decreased sequentially. (2) In the Structural Equation Model (*χ*^2 ^= 825.451, *p* < 0.001, df = 455, CMIN/df = 1.814, CFI = 0.942, RMSEA = 0.045), the three hypotheses of mediation were supported (*P* < 0.05), showing positive indirect effects between Physical Activity and Subjective Well-being. Of the three mediating effects, Social Support and Self-esteem were not different, and the mediating effect of Perceived Health showed the largest impact. This indicates that Social Support, Perceived Health, and Self-esteem mediate the effects of Physical Activity, and Subjective Well-being regulation has positive indirect effects.

**Conclusion:**

This study demonstrates the importance of meeting the needs of Social Support, Perceived Health, and Self-esteem when designing interventions to promote college students' sports participation to enhance Subjective Well-being.

## 1. Introduction

Most college students face mental health problems such as depression and anxiety (1, 2). In the United States, almost half of college-aged individuals have a psychiatric disorder (3). The prevalence of mental health problems among U.S. college students was higher in 2011 than in 2008, with 34.5% of college students reporting depressive symptoms according to the 2015 National College Health Assessment (4). Likewise, more than 20% of Chinese college students suffer from depression, and this ratio has continued to grow over the past decade (5). Research indicates that approximately half of university students have moderate stress-related mental health concerns, including anxiety and depression (6). Physical activity (PA), as a modifiable health behavior, has been identified as an influential factor in promoting physical and mental health (7). Regular participation in PA is associated with a reduced risk of cardiovascular disease, hypertension, type 2 diabetes, and depression (8). Mental health research should study positive psychological traits such as subjective well-being (SWB) (9). Despite evidence suggesting a positive impact of PA on mental health, PA has been primarily considered a method of preventing or treating mental disorders.

“The body of research on the link between PA and SWB is still in its early stages. SWB, according to Diener et al., is “a person's cognitive and affective evaluations of their life” (10). Living satisfaction is the overall judgment people make of their living situations (11, 12), and is the cognitive component of SWB, whereas positive and negative affect are the affective components (13). Happiness, defined as a subjective psychological state characterized by enjoyment and contentment, is extensively employed as a measure of SWB and an effective component of SWB (14). Life satisfaction is an essential variable since it is related to university students” mental and physical health and has been identified as one of the basic conceptions in the field of positive psychology (15). Thus, Understanding the factors that contribute to life satisfaction is a critical problem for university students. SWB dimensions can be mixed in a variety of ways, and SWB can be studied as a rapid cognitive assessment of one's health satisfaction.”

Perceived or self-rated health refers to an individual's perception of their health status. There is evidence that perceived health correlates more with SWB than objectively measured health (16, 17). From the perspective of perceived health, perceived health and well-being in adults are positively correlated among individuals and communities in the United States (18), and perceived health status is significantly associated with well-being in Sweden (19). Although objective health indicators such as physical health and functional rate have a relatively small effect on life satisfaction in older adults (20, 21), perceived health is significantly associated with well-being (22), and perceived health in middle-aged and older adults mediates PA and SWB (23). However, whether perceived health also mediates this relationship in college students remains unknown.

Social support is the feeling or experience of being loved, cared for, respected, and valued by a person as part of a social network of mutual aid and obligation (24). Social support is widely believed to be positively related to SWB (25, 26). Some studies suggest that social support is necessary for SWB (27). Social support should promote well-being by influencing cognition, emotion, and behavior to support positive affect (28). Social support varies by partner, family, and friends (29). Social help from family and friends positively relates to life satisfaction, but the interaction between these two variables is not statistically significant (30). Diener et al. (1999) (31) suggested that the theory must be refined to identify differential effects of input variables on components of SWB-specific predictions. There are separable components of SWB that exhibit unique patterns of relationships with different variables.

Self-esteem is an individual's overall sense of worth or value (Rosenberg, 1979) (32) and is framed in the context of demographic characteristics, social relationships, and personality (33–35). In many past studies, self-esteem has been strongly correlated with each component of SWB. Individuals with higher levels of self-esteem report higher life satisfaction and positive emotions and lower negative emotions, especially in individualistic cultures (33, 34, 36, 37). Self-esteem is strongly correlated with PA in children (38), and adolescents (39–41), and adults (42–44). Rosenberg et al. (1995) (45) emphasized that specific behaviors may be related to similar explicit self-esteem. In addition, it has been proposed that participation in PA contributes indirectly to an overall sense of self-worth and may enhance perceptions of the physical self, such as perceived physical appearance and physical motor ability (46).

In addition, several studies have shown that self-esteem is one of the strongest predictors of the cognitive component of SWB in adolescents and adults (47, 48). On the other hand, past research has shown that social support appears to have direct and indirect effects on well-being through specific cognitive mechanisms, personality factors (e.g., optimism, self-efficacy), and health behaviors (49–51). Considering that social support contributes theoretically (32, 52)and empirically (53–55) to self-esteem, self-esteem contributes theoretically and empirically to SWB.

The positive effects of PA on physical and mental health are well documented. Previous research has widely documented the positive relationship between PA and various physical health parameters (56–58). PA is associated with lower overall mortality (59), improved cardiovascular and musculoskeletal health (60), lower risk of obesity and stroke (59), lower mental health burdens (61), and reduced symptoms of depression and anxiety (62–64). Studies based on a large general population suggest a positive correlation between PA and SWB (65, 66), and some studies suggest that regular PA may increase SWB in all age groups: children and adolescents (15, 67); young adults (68); adults (69, 70); and older adults (71–73). Research on this relationship among college students is limited, and only a few relevant studies exist.

The findings reveal the relationship between PA and SWB but can only provide generalizability to a limited extent. Most recent well-being studies focus on the relationship between a single factor and SWB, lacking systematic integration of various influencing factors, yet multiple factors influence SWB simultaneously. Only by considering individual factors, i.e., external environmental factors, can a multidimensional integration model be formed to explore the direct or indirect effects and contributions of individual factors on well-being. In addition, perceived health, social support, and self-esteem positively impact psychological well-being. In conclusion, given the robust evidence of a strong relationship between health and SWB and the beneficial effects of PA on health, we conclude that the relationship between PA and SWB among university students may be mediated by perceived health, social support, and self-esteem.

Research on the mediating variables of PA and SWB among the areas of university students, PA and Perceived Health, Social Support, and Self-esteem has become a significant research topic. This study uses the Physical Activity Scale, SWB Scale, Perceived Health Scale, Social Support Scale, and Self-esteem to analyze the effects of PA on university students' SWB and to explore whether Perceived Health, Social Support, and Self-esteem mediate the effects of PA on SWB, taking Chinese university students, a particular group, as its sample.

## 2. Method

The purpose of this study was to investigate the association between PA and SWB among university students, as well as the function of Perceived Health, Social Support, and Self-esteem in mediating the relationship between PA and SWB. As a result, the research subjects, research equipment, and data processing were carried out in accordance with the study's purpose.

### 2.1. Research model and research hypothesis

#### 2.1.1. Research model

**Figure 1:**
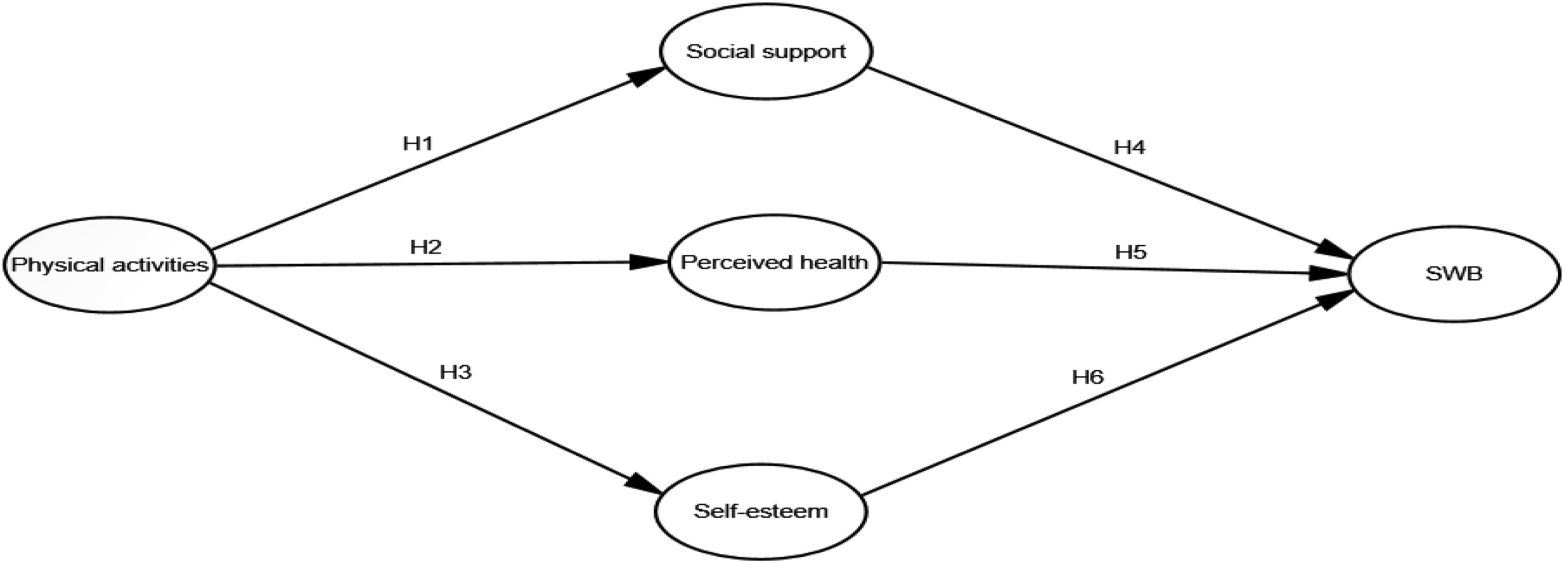
Research model.

As shown in Figure 1, this study aimed to determine the effect of PA on university students' SWB and explore whether Perceived Health, Social Support, and Self-esteem mediate the effect of PA on SWB.

#### 2.1.2. Research hypothesis

According to the research model and the problems to be solved, we put forward nine corresponding research hypotheses, which are described as follows:

H1 Physical activity affects social support.
H2 Physical activity affects perceived health.
H3 Physical activity affects self-esteem.
H4 Social support affects subjective well-being.
H5 Perceived health affects subjective well-being.
H6 Self-esteem affects subjective well-being.
H7 Social support mediates the relationship between physical activity and subjective well-being.
H8 Perceived health mediates the relationship between physical activity and subjective well-being.
H9 Self-esteem mediates the relationship between physical activity and subjective well-being.

### 2.2. Participants

Participants for this study were recruited through Questionnaire Star, and 420 Chinese university students were identified from May to June 2022. An explanation of the primary purpose and content of the survey was given to participants in Questionnaire Star, and written informed consent was obtained from all participants before the survey. The study protocol was approved by the Institutional Review Board (IRB)-(1040875-202202-SB-021) in Korea. These participants completed questionnaires assessing their well-being, life satisfaction, PA, and perceived health during the assessment. As reported in Table 1, a total of 420 questionnaires were recovered, and those who skipped more than 16 questions or answered the questionnaire incorrectly were excluded, leaving 404 samples for further analysis. Looking at the individual characteristics in detail, 147 males (36.4%) and 257 females (63.6%) were included. As indicated previously, the survey captured College year groups: Freshman (28, 6.9%); Sophomore (141, 34.9%); Junior (143, 35.4%); Senior (92, 22.8%). In terms of Habit, less than 12 months (304, 75.2%), more than 12 months (100, 24.8%).

**Table 1:** Socio-demographic characteristics of participants (*N* = 404).

Characteristics		Number (persons)	percent (%)
Sex	Male	147	36.4
	Female	257	63.6
Age	18	5	1.2
	19	47	11.6
	20	88	21.8
	21	78	19.3
	22	75	18.6
	23	62	15.3
	24	31	7.7
	25	10	2.5
	26	6	1.5
	27	2	0.5
College year	Freshman	28	6.9
	Sophomore	141	34.9
	Junior	143	35.4
	Senior	92	22.8
Habits	0–3 months	166	41.1
	3–6 months	95	23.5
	6–12 months	43	10.6
	12–18 months	18	4.5
	More than18 months	82	20.3

### 2.3. Measures

The Physical Activity Scale, Social Support Scale, and SWB, and Perceived Health, and Self-esteem measures were employed in this study. We investigated the effect of PA on college students' SWB using a cohort of Chinese university students as subjects. We investigated whether perceived health, social support, and self-esteem act as moderators of the effect of PA on SWB.

#### 2.3.1. Moderate to vigorous physical activity (MVPA)

MVPA was measured using two questions adapted from the PA and physical fitness section of the 2007–2008 National Health and Nutrition Examination Survey (74):(1) “In a typical week, how much time do you usually spend doing moderate-intensity physical activities that cause small increases in breathing or heart rate?” and (2) “In a typical week, how much time do you usually spend doing vigorous-intensity physical activities that cause large increases in breathing or heart rate?” (75, 76).

#### 2.3.2. Subjective well-being scale

##### 2.3.2.1. Positive affect and negative affect scale

Positive affect and negative affect were assessed using the Chinese-translated version of the Scale of Positive and Negative Experience (SPANE) (77). The validity and reliability of SPANE have been tested previously among Chinese adults (78). The SPANE has twelve items describing positive and negative feelings such as “pleasant,” “joyful,” and “sad.” The respondents were asked to rate the extent to which they experienced each item using a 5-point Likert scale from 1 (very rarely or never) to 5 (very often or always). The SPANE produces a score for positive affect (Cronbach's Alpha = 0.914) and a score for negative affect (Cronbach's Alpha = 0.901) by adding the scores of the corresponding items.

##### 2.3.2.2. Happiness scale

Happiness was measured as the affective component of SWB using the Chinese translated version of the Subjective Happiness Scale (SHS) (79). The SHS consists of four items on happiness, asking to what extent you agree or disagree with statements including (1) “In general, I consider myself a very happy person,” and (2) “Compared to most of my peers, I consider myself happier.” Response options were scaled from 1 (strongly disagree) to 7 (strongly agree). In this study, Cronbach's alpha coefficient for the Happiness Scale index was 0.871.

##### 2.3.2.3. Life satisfaction scale

Life satisfaction was measured as the cognitive component of SWB using the Chinese translated version of the Satisfaction with Life Scale (SWLS) (80). The SWLS consists of five items to measure global cognitive judgments of life satisfaction, including (1) “In most ways, my life is close to my ideal,” (2) “The conditions of my life are excellent,” (3) “I am satisfied with life,” (4) “So far, I have gotten the important things I want in life,” and (5) “If I could live my life over, I would change almost nothing.” The score of each item ranges from 1 (strongly disagree) to 7 (strongly agree), indicating how much you agree or disagree with each statement. A mean score was computed by averaging the score of each item, with higher scores indicating greater life satisfaction. Previous studies have shown that the SWLS has good levels of reliability and validity for Chinese university students (81, 82). In this study, Cronbach's alpha coefficient for the Life Satisfaction Scale index was 0.879.

#### 2.3.3. Perceived health scale

Perceived health was assessed using a single item: “During the past 30 days, how often did you feel very healthy and full of energy?” The predefined responses were “never,” “seldom,” “sometimes,” “oftentimes,” and “always” (coded as 1–5). This one-item question was adapted from the healthy days core module of the 2001 Behavioral Risk Factor Surveillance System (BRFSS) survey (83, 84).

#### 2.3.4. Social support scale

To assess perceived social support in participants, we administered the Multi-Dimensional Scale of Perceived Social Support (MSPSS) (85), consisting of twelve items. The scale assesses three sources of support: significant other, family, and friends. Example items include statements like “There is a special person who is around when I am in need”, “There is a special person with whom I can share my joys and sorrows”, and “My family really tries to help me”. Each item is answered on a seven-point Likert scale ranging from 1 = strongly disagree to 7 = strongly agree. In this study, Cronbach's alpha coefficient for the Social Support Scale index was 0.865.

#### 2.3.5. Self-Esteem scale

The Rosenberg Self-Esteem Scale contains ten items scored on a four-point Likert scale and provides an overall evaluation of one's worth or value (Rosenberg, 1965) (86). For example, items included “I feel that I have a number of good qualities,” “I feel I do not have much to be proud of,” and “I feel that I’m a person of worth, at least on an equal plane with others.” In this study, Cronbach's alpha coefficient for the Self-Esteem Scale index was 0.811.

### 2.4. Statistical analysis

In this study, the descriptive statistics and reliability of the demographic characteristics of the study population were studied using SPSS 23.0, and correlation analysis was performed using SPSS 23.0. The AMOS 24.0 program was used to perform validation factor analysis, construct models, etc. The main research questions and analysis were conducted in the following ways.

First, the questions were subjected to a validation factor analysis to filter out the questions suitable for representing each dimension and verify the validity of the measurement instrument. Second, Cronbach's alpha values were calculated to verify the reliability of each item of the questionnaire, such as the tools from the measurement scales for PA, SWB, perceived health, social support, and self-esteem. Third, correlation analysis was conducted using the correlation coefficient of Person to identify problems of multicollinearity in the observed variables. Fourth, descriptive statistical analysis was conducted to understand the demographic characteristics of each measured variable. Fifth, the direct effects in the model were analyzed by using structural equation modeling to predict the variables. Sixth, the PA factor of college students was used as the independent variable, the SWB factor was used as the dependent variable, and the perceived health, social support, and self-esteem of college students were used as mediating variables. The significance of indirect effects was verified by bootstrapping. All statistical tests set the statistical significance level at *p* < 0.05.

## 3. Results

In this study, AMOS 24.0 was used to construct structural equation models before structural modeling analysis to test the proposed hypotheses. Confirmatory factor analysis (CFA) was conducted first, followed structural equation modeling (SEM) estimation according to Anderson and Gerbing's (1988) (87) two-step approach and recommended principles. The consistency of all scales was assessed, with a high Cronbach's alpha of 0.70 indicating good internal consistency (Fomell & Lacker, 1981) (88). In addition, this study used *χ*^2^, df, CMIN/DF, CFI, and RMSEA to further analyze the model's fit to the data, with CMIN/DF less than 3, CFI greater than 0.9, and RMSEA less than 0.08.

### 3.1. Structural model

**Figure 2:**
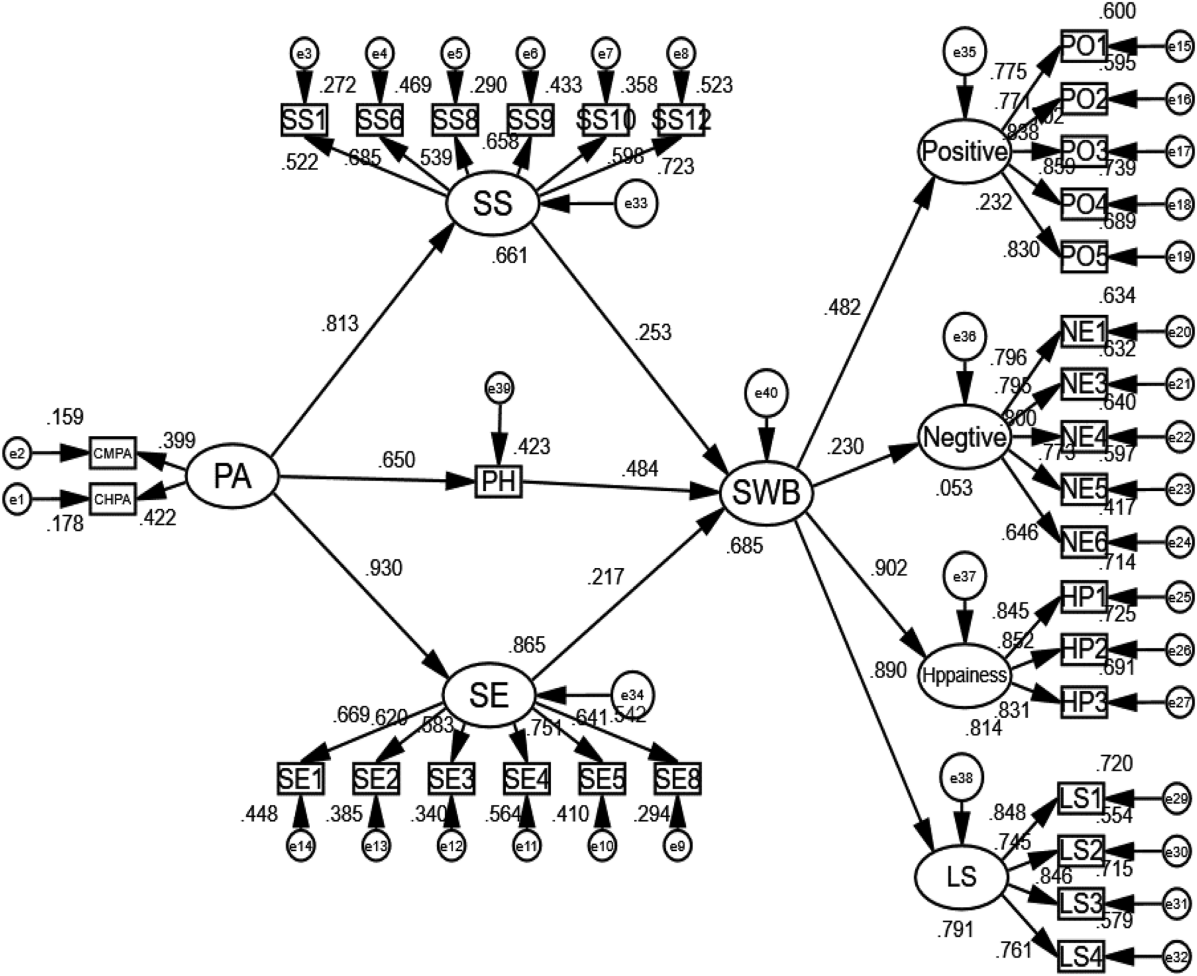
The result of the structural model.

AMOS 24.0 was used to establish the structural equation model and test the hypotheses based on the theoretical model and hypotheses proposed in the study. Table 2 shows that the fitness of the structural equation model of the complete model is *χ*^2 ^= 825.451, *p* < 0.001, df = 455, CMIN/df = 1.814, CFI = 0.942, RMSEA = 0.045. The fitness of the structural equation model can be evaluated as good. The structural equation model analysis result is shown in Figure 2.

**Table 2:** The result of the structural model.

CMIN	DF	CMIN/DF	CFI	RMSEA
825.451	455	1.814	0.942	0.045

Table 3 show the results of hypothesis testing.

**Table 3:** The structural model analysis result.

Path		Estimate	SE	Z	*P*	*β*	*R* ^2^	Results
H1	PA→SS	0.262	0.045	5.851	***	0.813	0.661	support
H2	PA→PH	0.513	0.075	6.874	***	0.650	0.423	support
H3	PA→SE	0.354	0.058	6.127	***	0.930	0.865	support
H4	SS→SWB	0.257	0.087	2.934	0.003	0.253	0.685	support
H5	PH→SWB	0.200	0.029	6.841	***	0.484		support
H6	SE→SWB	0.186	0.077	2.411	0.02	0.217		support

PA, Physical Activities; SS, Social Support; PH, Perceived Health; SE, Self-Esteem; SWB, Subjective Well-Being; *χ*^2 ^= 825.451, *p* < 0.001, df = 455, CMIN/df = 1.814, CFI = 0.942, RMSEA = 0.045; 3.

*** *P *< .001.

The path coefficient of the effect of PA on social support was significantly positive, with a path coefficient of 0.813; hypothesis H1 was supported.

The path coefficient of the effect of PA on perceived health was significantly positive, with a path coefficient of 0.650; hypothesis H2 was supported.

The path coefficient of the effect of PA on self-esteem was significantly positive, with a path coefficient of 0.930; hypothesis H3 was supported.

The path coefficient of the effect of social support on SWB was significantly positive, with a path coefficient of 0.253; hypothesis H4 was supported.

The path coefficient of the effect of perceived health on SWB was significantly positive, with a path coefficient of 0.484; hypothesis H5 was supported.

The path coefficient of the effect of self-esteem on SWB was significantly positive, with a path coefficient of 0.217; hypothesis H6 was supported.

The R^2^ value was used to analyze the predictive power of each variable. The values should be sufficiently high for the model to have a minimum level of explanatory power. Chin (1998) (89) considers values of approximately 0.670 substantial, approximately 0.333 average, and values of 0.190 and lower weak. PA accounted for 66.1% of the variance in social support (*R*^2^ = .661). PA was 42.3% of the variance in perceived health (*R*^2^ = .423) and 86.5% of the variance in self-esteem (*R*^2^ = .865). Social support for SWB, perceived health, and self-esteem explained 68.5% of the variance in SWB (*R*^2^ = .685).

### 3.2. Mediation effect

To verify the mediation effect more accurately, the bootstrap method is adopted. The data were bootstrapped with repeated sampling 5,000 times, with the confidence interval level set at 95%, and the sampling method was a nonparametric percentile with deviation correction.

As seen in Table 4, the indirect effect of PA→SS→SWB was 0.067 with a 95% confidence interval (.019-.164), excluding zero, indicating a significant mediating impact. The indirect effect of PA→PH→SWB was 0.103, with a 95% confidence interval (.057-.180), excluding zero, marking a significant mediating effect. The immediate impact of PA→SE→SWB was 0.066, with a 95% confidence interval (.009-.172), excluding zero, indicating a significant mediating effect.

**Table 4:** Mediation test.

Path		Estimate	Product of Coefficients		Bootstrapping		
					BC 95% CI		Two-tailed significance
			SE	Z	Lower	Upper	
H7	PA→SS→SWB	0.067	0.035	1.914	0.019	0.164	0.005 (***)
H8	PA→PH→SWB	0.103	0.030	3.433	0.057	0.180	0 (***)
H9	PA→SE→SWB	0.066	0.041	1.610	0.009	0.172	0.026 (**)

PA, Physical Activities; SS, Social Support; PH, Perceived Health; SE, Self-Esteem; SWB, Subjective Well-Being; *χ*^2 ^= 825.451, *p* < 0.001, df = 455, CMIN/df = 1.814, CFI = 0.942, RMSEA = 0.045; 3.

** *P *< .01

*** *P *< .001.

## 4. Discussion

There is much research on PA and SWB, and many studies and meta-analyses report a close relationship between the variables of PA and SWB (66). According to Zhang and Chen's (4) recommendations, the researchers combine PA with SWB by leveraging their theoretical advantages for describing health to improve their interpretations through the mediating effects of perceived health, and social support, and self-esteem. Predictions were made through a holistic model that promotes approach- and reality-based intervention plans. This study attempted to address the following areas: To examine the relationship between PA and the SWB of university students, and to observe whether perceived health, social support, and self-esteem played a mediating role on the relationship between PA and SWB.

This study helps to elucidate the effect of PA on SWB. The results showed that (1) according to the measurement model, all hypotheses were supported by the results. PA was related to social support, perceived health, and self-esteem; social support, perceived health, and self-esteem were related to SWB. However, the direct effect of PA observed in H1-H3 became more minor in self-esteem, social support, and perceived health. The direct effects of perceived health, social support, and self-esteem on SWB decrease sequentially. (2) According to the structural model, all three mediating hypotheses were supported. All three mediating effects showed positive indirect effects between PA and SWB. Of the three mediating effects, social support and self-esteem were not different, and the mediating result of perceived health had the greatest effect. This indicates that social support, perceived health, and self-esteem mediate PA to positively affect SWB regulation.

Studies based on a large general population have shown a positive correlation between PA and SWB and that PA is a crucial predictor of life satisfaction (90), consistent with the results of college students in this study (65, 66). It is widely accepted that social support positively relates to SWB (25, 26). Some studies have even shown that social support is necessary for SWB, consistent with the results of the college students in this study (27).

Direct impact studies in research have shown that self-esteem explains PA better than social support and perceived health, with PA contributing less to predictions of perceived health. Self-esteem was one of the strongest predictors of the cognitive component of SWB in adolescents and adults, which is consistent with the results of previous studies (35, 47, 48). Perceived health better explained SWB, with similar levels of prediction for social support and self-esteem. Perceived health was more strongly correlated with SWB than objectively measured health (16, 17), similar to previous studies. When social support, perceived health, and self-esteem were used as mediating variables, they positively influenced PA and SWB, with all three mediating effects significantly moderated. Analysis of the pathway results indicated that perceived health was the best mediator of the mediating effect. Past research has shown that social support appears to have a direct and indirect impact on wellbeing through specific cognitive mechanisms, personality factors, and health behaviors (26, 49–51). Perceived health in middle-aged and older adults mediates PA and SWB (23). This study demonstrated this relationship is mediated by perceived health also among college students.

The present study has important theoretical implications for research related to PA and SWB. In this paper, we systematically review the literature on PA and SWB to identify as many factors that affect them as possible and to provide a more comprehensive understanding of the research in this field. Some scholars have studied the relationship between PA and SWB in the past, and some studies have separately modeled the three dimensions of social support, perceived health, and self-esteem, but no scholars have developed a comprehensive structural model of social support, perceived health, and self-esteem along with PA and SWB to explore their relationship. In this paper, we explore the relationship between PA and SWB among Chinese university students through a web-based survey and verify the mediating effects of social support, perceived health, and self-esteem.

From a practical perspective, this study provides guidance for future scholars who study PA and SWB. First, based on the final results of the mediated effects, we will increase the attention brough to social support, perceived health, and self-esteem in future PA interventions for college students to enhance physical fitness and improve SWB at the same time. Second, in the context of enhancing SWB, this study strengthens the capacity of physical education teachers to give guidance to students to mobilize their own pursuits, changing traditional teaching concepts to keep up with the times, and improving the developmental consciousness of students in a comprehensive manner to better provide precise services for Chinese university sports participants.

Despite the limitations of the study, the findings are valuable. The study discovered a mediation influence between PA and SWB in terms of social support, perceived health, and self-esteem. However, there are some restrictions, most notably in the following areas: (1) To begin, the study employs a cross-sectional design, which gives a measure of past or current behavior rather than a prediction of future conduct. Because of its limitations, consistency bias may exist, making it impossible to discern causal links between variables. Longitudinal designs and randomized controlled trials may be used in future studies to assist in demonstrate causal links between SWB and PA. (2) One weakness of this study is the use of self-report tools. Because of the assessor, the context of measurement, and the content and qualities of the question items, such self-reporting can lead to changes in independent variables and changes in artifacts across variables, all of which can lead to differing participant reports. Thus, despite efforts to control for ambiguity in the question items, scale format, and questionnaire length, some uncontrollable elements in the study, such as societal expectations and subject response emotions, may have nevertheless influenced the outcomes. (3) Because this study was conducted with Chinese university students, it is crucial to exercise caution when extrapolating the findings to other populations.

## 5. Conclusion

This study focused on determining the relationship between physical activity and subjective well-being among Chinese university students, and the mediating effects of social support, perceived health, and self-esteem was verified in the composed integrated theoretical model. For application in physical activity research, we emphasized the role of social support, perceived health, and self-esteem in physical activity and subjective well-being based on evidence from previous studies, with theory-guided research promoting a more profound understanding among researchers and research subjects. Thus, the current study provides a valuable framework for research related to physical activity and subjective well-being. Finally, our findings suggest that creating a psychological environment that satisfies social support, perceived health, and self-esteem is essential to encouraging college students to participate in sports. This study theoretically confirms that interventions in the areas of social support, perceived health, and self-esteem ultimately results in enhancing college students' subjective well-being and quality of life and reducing conditions such as stress and depression.

## Data Availability

The raw data supporting the conclusions of this article will be made available by the authors, without undue reservation.
